# Phytochemical-Based Therapeutic Strategies for Sarcopenia: From Molecular Mechanisms to Clinical Translation

**DOI:** 10.3390/ph19060905

**Published:** 2026-06-07

**Authors:** Gengyun Le-Chan, Nicole Q. Amoah, Hailey M. Sofia, Aidan H. Quee, Sunny S. K. Chan, Cindy A. Thomas-Charles

**Affiliations:** 1 Department of Biology, Hillyer College of Arts & Sciences, University of Hartford, 200 Bloomfield Avenue, West Hartford, CT 06117, USA; 2 Department of Health Sciences, College of Education, Nursing, and Health Professions, University of Hartford, 200 BloomfieldAvenue, West Hartford, CT 06117, USA; 3 Department of Rehabilitation Sciences, College of Education, Nursing, and Health Professions, University of Hartford, 200 Bloomfield Avenue, West Hartford, CT 06117, USA; 4 Department of Math and Sciences, Connecticut State Community College Norwalk, 188 Richards Avenue, Norwalk, CT 06854, USA

**Keywords:** phytochemical, muscle, sarcopenia, aging, pharmaceutical

## Abstract

Sarcopenia is a progressive, age-related musculoskeletal disorder characterized by the loss of skeletal muscle mass, strength, and physical performance, which contributes to frailty, disability, and mortality in older adults. Although resistance exercise and optimized protein intake remain first-line interventions, effective pharmacological therapies are limited, highlighting the need for novel adjunctive strategies. Increasing interest has focused on phytochemicals, plant-derived bioactive compounds with antioxidant, anti-inflammatory, and metabolic regulatory properties that may target multiple mechanisms underlying muscle aging. This review summarizes the molecular and translational potential of phytochemicals in sarcopenia management. Experimental and emerging clinical evidence indicates that flavonoids, polyphenols, alkaloids, and terpenoids modulate key pathways involved in sarcopenia pathogenesis, including PI3K/Akt/mTOR-mediated anabolic signaling, AMPK–SIRT3–PGC-1α-dependent mitochondrial biogenesis, NF-κB-driven inflammation, oxidative stress responses, autophagy, and satellite cell function. Through these pleiotropic effects, phytochemicals may attenuate the anabolic resistance, mitochondrial dysfunction, chronic inflammation, and impaired muscle regeneration associated with aging. Despite promising mechanistic evidence, clinical translation remains limited by poor bioavailability, variability in formulation and dosing, a lack of long-term randomized trials, and inconsistent functional outcome measures. Current evidence suggests that phytochemicals are most effective when integrated with resistance exercise and nutritional support rather than used as stand-alone therapies. Overall, phytochemicals represent promising complementary candidates for sarcopenia prevention and management. Future studies should prioritize standardized formulations, biomarker-guided approaches, and rigorously designed clinical trials focused on clinically meaningful functional outcomes to establish their efficacy, safety, and translational relevance in aging populations.

## 1. Pathophysiology of Sarcopenia

Sarcopenia is a progressive and generalized skeletal muscle disorder characterized by an age-related decline in skeletal muscle mass (myopenia) [1], strength (dynapenia) [2], and physical performance (frailty) [1,2,3,4]. Consistent with the consensus framework established by international working groups, including the European Working Group on Sarcopenia in Older People (EWGSOP) and the Rome consensus definition, sarcopenia is recognized as a multifactorial geriatric syndrome, involving both quantitative and functional deterioration of skeletal muscle. The condition is driven by complex interactions among aging, physical inactivity, endocrine alterations, chronic inflammation, insulin resistance, nutritional deficiencies, and chronic disease states. The present review primarily focuses on primary (age-related) sarcopenia and the molecular mechanisms underlying its progression. However, because many pathogenic pathways overlap with secondary or disease-associated sarcopenia, including inflammation, mitochondrial dysfunction, and anabolic resistance, selected evidence from chronic disease models and translational studies is also discussed where relevant to phytochemical-based therapeutic interventions.

One of the central features of sarcopenia is anabolic resistance, in which aging skeletal muscle exhibits a diminished response to anabolic stimuli such as insulin, insulin-like growth factor-1 (IGF-1), amino acids, and exercise [5]. The suppression of insulin/IGF-1 signaling and the dysregulation of nutrient-sensing pathways, including AMP-activated protein kinase (AMPK), impair protein synthesis while promoting proteolysis [6,7]. Concurrent activation of catabolic mediators such as myostatin and Nuclear Factor κB (NF-κB) further accelerates muscle protein degradation and myofiber atrophy [8].

Chronic low-grade inflammation (“inflammaging”) also contributes substantially to sarcopenia progression. Elevated circulating inflammatory mediators, including IL-6, TNF-α, and C-reactive protein (CRP), activate catabolic signaling cascades and exacerbate muscle wasting [9,10,11]. These inflammatory processes are further amplified by age-associated cellular senescence and altered intercellular communication, which promote tissue degeneration and impair muscle repair.

Mitochondrial dysfunction represents another major pathogenic mechanism. Aging muscle demonstrates impaired mitochondrial biogenesis, defective mitophagy, the accumulation of mitochondrial DNA damage, and increased oxidative stress [11,12,13]. These alterations compromise cellular bioenergetics, increase reactive oxygen species production, and contribute to insulin resistance and inflammatory activation, thereby reinforcing anabolic failure and muscle degeneration.

Neuromuscular decline further impairs muscle quality and functional performance. Age-related motor neuron loss, defective neuromuscular junction signaling, reduced vascular perfusion [14,15,16], extracellular matrix remodeling [17,18], and ionic dyshomeostasis collectively compromise excitation–contraction coupling and decrease muscle strength independently of muscle mass loss.

Furthermore, impaired regenerative capacity contributes to the progressive nature of sarcopenia. Hallmarks of aging, including genomic instability, epigenetic alterations, telomere attrition, loss of proteostasis, and stem cell exhaustion, compromise satellite cell function and skeletal muscle regeneration [9,10]. These intrinsic aging mechanisms interact with extrinsic factors such as physical inactivity, malnutrition, hormonal decline, chronic disease, and lifestyle-related stressors to accelerate disease progression [19,20]. Collectively, the interconnected mechanisms shift muscle homeostasis toward net protein degradation, myofiber atrophy, and functional decline, highlighting multiple convergent therapeutic targets for pharmacological and multimodal interventions in aging-related musculoskeletal disorders.

Relevant literature for this narrative review was identified through searches of PubMed, Google Scholar, Web of Science, and MEDLINE databases for articles published between 2000 and 2026. The search strategy included combinations of the following keywords and related terms: “phytochemical,” “sarcopenia,” “skeletal muscle,” “muscle aging,” “aging,” and “pharmaceutical.” Priority was given to peer-reviewed experimental, translational, and clinical studies examining the molecular mechanisms and therapeutic potential of phytochemicals in sarcopenia and age-related muscle dysfunction.

## 2. Phytochemicals: Definition and Classification

Phytochemical is among several terms used to describe plant-derived compounds with demonstrated biological activity. Many of these plant-derived compounds are classified as secondary metabolites since they do not directly contribute to basic metabolic processes. Instead, they play critical roles in plant growth, defense against pathogens and pests, and adaptation to environmental stressors (Table 1). While phytochemicals are a broad group of compounds, they are subdivided into chemical families based on their structure and biological function [21]. The major subdivisions of phytochemicals include phenols, terpenes, organosulfur compounds, alkaloids, phytosterols, and betalains, which can be found in a wide variety of edible plants such as fruits, vegetables, nuts and grains [22]. In recent decades, phytochemicals have attracted significant scientific interest due to their potential health-promoting properties. Numerous studies provide evidence of their potential to reduce the risk of chronic diseases including cancer, diabetes, and aging-related illnesses. These beneficial effects have been attributed to the antioxidant, anti-inflammatory, and signal-modulating activities of phytochemicals [23,24].

### 2.1. Polyphenols

These are the most abundant and most well-studied groups of phytochemicals and are valued for their antioxidant properties. Structurally, members of this subgroup contain at least one aromatic ring conjugated to one or more hydroxyl groups. These bioactive compounds are abundant in fruits and vegetables and are further subdivided into classes that include flavonoids, phenolic acids, and stilbenes [48]. The polyphenolic structure forms four subclasses of flavonoids: flavones, flavanols, flavanones, and flavanonols, each exhibiting distinct health benefits. There is a growing recognition that flavonoids are beneficial for maintaining muscle health in the context of age-related muscle atrophy. Of these classes, flavonoids are among the most well-explored and are further subdivided into flavonols, flavones, isoflavonoids, flavanols, flavanones, and anthocyanidins [49]. There is a growing body of evidence that establishes the biological activity of flavonoids beyond their antioxidant properties to include modulation of inflammatory and cytoprotective pathways, cellular proliferation, anti-neurodegeneration and anti-microbial defense [50,51,52,53]. The benefits of polyphenols are largely determined by their bioavailability, which in turn depends on the interaction of these bioactives with other dietary components, including macronutrients [54,55].

### 2.2. Terpenes

Also known as terpenoids, terpenes are a large and structurally diverse class of lipid-soluble pigments synthesized by plants, algae, and some microorganisms. Terpenes contain five-carbon isoprene units and are categorized based on the number of these units they contain. They are essential constituents of cells with roles in important processes including metabolism and signal transduction [56]. A key and well-explored subclass of terpenes is the carotenoids, which include α and β carotene, lycopene, and lutein [22]. Carotenoids are 40-carbon-based iosoprenoid pigments that have roles in the modulation of inflammation and oxidative stress pathways [57]. They are responsible for the red, orange, and yellow coloring of foods such as carrots, sweet potatoes, tomatoes, leafy greens, and brightly colored fruits. As with other phytonutrients, the biological activity of carotenoids is strongly dependent on the absorption and bioavailability of the compounds [58,59,60,61].

### 2.3. Organosulfur Compounds

These compounds are organic molecules that contain a sulfur atom bonded to a carbon atom. They are a defense mechanism produced as a deterrent to herbivorous insects. They are found in a variety of fruits, vegetables, and mushrooms where they are stored conjugated to other molecules to form larger compounds that can be readily activated following cellular damage. Well explored organosulfur compounds such as allicin have anti-cancer, anti-inflammatory, antibacterial, and antifungal activities that are based on the action of metabolizing enzymes and sequestration of reactive oxygen species [62,63,64].

### 2.4. Alkaloids

These are a large and diverse family of phytochemicals that are mainly derived from amino acid precursors and classified as either heterocyclic or non-heterocyclic based on the position of their nitrogen atoms [65]. Alkaloids have been identified and isolated from a variety of sources including plants and fungi, where they function as metabolites [66]. Similar to organosulfur compounds, alkaloids often form larger scaffolded complexes with other molecules, a feature that influences their classification [67]. Many common alkaloids such as caffeine, nicotine, and capsaicin function as stimulants and can modulate metabolism [68,69], pain perception [70], and preservation of skeletal muscle [71].

### 2.5. Phytosterols

These compounds are structurally and functionally related to cholesterol. More than 200 phytosterols have been identified in a variety of plant products including oils, nuts, and seeds. In plants, thein main functions include supporting the normal function and structure of cellular membranes. These compounds compete with cholesterol for absorption in the gut and as such have been found to have the capacity to reduce blood pressure and the risk of cardiovascular disease [72,73,74,75]. Additionally, studies show that phytosterols, which have the ability to cross the blood–brain barrier, can modulate signaling pathways implicated in the development of neurodegenerative diseases [76]. Furthermore, evidence of a strong correlation between the capacity of phytosterols to regulate gut microbiota has been found to have a significant impact on the development of glucocorticoid-induced osteoporosis [77].

### 2.6. Betalains

These are water-soluble nitrogen-containing compounds that are known for their vibrant pigmentation. Betalains are present in a variety of fruits and vegetables, most notably beets, from which the name derives, and the prickly pear cactus [78,79,80]. These compounds are derived from tyrosine precursors and are subdivided into betacyanins (red-violet pigments), and betaxanthins (yellow-orange pigments) [78]. These compounds are best known for their antioxidant and anti-inflammatory activities [78,81,82].

## 3. Polyphenols with Therapeutic Potential in Sarcopenia 

**Figure 1:**
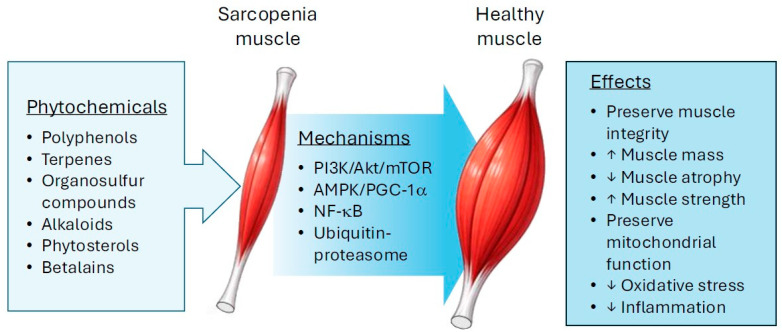
Potential molecular mechanisms of phytochemicals contribute to the therapy and management of sarcopenia.

Increasing evidence suggests that polyphenols may play a protective role against sarcopenia, the age-related decline in skeletal muscle mass and function [83,84]. Mechanistically, polyphenols exhibit antioxidant and anti-inflammatory properties that can mitigate oxidative stress and chronic low-grade inflammation, two major contributors to muscle aging [85,86]. In addition, polyphenols may enhance mitochondrial function and regulate key signaling pathways involved in muscle protein turnover, including mTOR, AMPK, and NF-κB [87,88]. Through these mechanisms, polyphenols may promote anabolic signaling, reduce protein degradation via the ubiquitin–proteasome pathway, and improve overall muscle metabolic health (Figure 1).

Experimental and clinical studies have begun to support the potential benefits of polyphenols for muscle preservation. For example, the green tea catechin epigallocatechin-3-gallate (EGCG) has been shown in animal models to increase muscle mass and reduce the expression of proteolytic markers such as MuRF1 and MAFbx while enhancing anabolic factors including IGF-1 and IL-15 [83,89]. Other polyphenol sources, including grape-derived compounds, marine oligomeric polyphenols, and litchi-derived oligonol, have demonstrated improvements in muscle-related outcomes through mechanisms involving enhanced mitochondrial quality, increased nutrient signaling, and modulation of amino acid metabolism. Furthermore, polyphenol-rich foods and supplements may influence gut microbiota composition, leading to increased production of short-chain fatty acids and improved antioxidant enzyme activity, which may indirectly support muscle health. Additionally, flavonoids, including isoorientin and isoschaftoside, promote myogenic differentiation by upregulating myogenic regulatory factors such as MyoD and myogenin [90].

Evidence from human studies remains limited but promising. A recent meta-analysis of interventional trials in sarcopenic individuals reported a significant improvement in muscle mass following polyphenol supplementation, although no significant effect was observed for muscle strength and only a trend toward improved physical performance was noted. Collectively, these findings suggest that polyphenols may represent a valuable adjunct nutritional strategy for mitigating muscle loss during aging. However, larger and longer-term clinical trials are required to clarify their effectiveness and establish optimal dosing strategies.

### 3.1. Isoflavone

Isoflavones are estrogen-like flavonoids found in soy [31]. Fermentation of soy results in the formation of isoflavone aglycones including genistein, daidzein, and glycitein [31]. In females, the age-related decline in estrogen is associated with an increased risk of developing sarcopenia [91]. In a recent study, the effects of isoflavones on sarcopenia were studied in ovariectomized mice. There was a total of 12 female mice that were then separated into two separate groups: control (*n* = 6) and isoflavone (*n* = 6) group [31]. The control group was fed a high-fat, high-sucrose diet with normal water, and the isoflavone group was fed the same diet but with soy isoflavone water; this treatment was tested for six weeks [31]. The soy isoflavones utilized in this experiment had an isoflavone content of 40.74%, with the most abundant isoflavones being malonyl daidzin and daidzin [31]. The results indicated that the isoflavone-treated mice had higher grip strength, the soleus muscle displayed decreased Trim63 expression, and there was an inactivation of muscle-specific RING finger protein 1 [31]. The results of this study suggest that the intake of isoflavones in mice with sarcopenia can suppress TNF-α signaling via NF-κB and the expression of the unfolded protein response, which in turn is suspected to inhibit muscle atrophy [31].

### 3.2. Sinensetin

Sinensetin is a citrus-derived polymethoxyflavone [32]. The presence of multiple methyl groups is a defining feature of this compound and contributes to its biological activity [92]. To test the effect of sinensetin on sarcopenia, a current ex vivo study collected muscle cells from the thigh and calf tissues of young (6-week-old) and old (12-month-old) rats [32]. After the collection of the skeletal muscle tissue, the isolation and differentiation of satellite cells were completed, followed by an analysis of morphological changes and the measurement of myoblast length [32]. It was determined that the older rats were experiencing muscle loss; however when the cells of the old rats were treated with 50 and 100 μM of sinensetin for five consecutive days, their diameter and length increased [32]. Additionally, it was observed that the protein expression levels of myoD and myogenin were upregulated [32]. Overall, it can be determined that sinensetin has a promising effect on age-related sarcopenia in relation to cell differentiation and the protein levels of myoD and myogenin.

### 3.3. Quercetin

Quercetin is an antioxidant flavonoid found in onions, cabbage, tomatoes, and leafy vegetables [93]. This flavonoid has shown promising effects against dexamethasone-induced skeletal muscle atrophy caused by the use of glucocorticoids [93]. Glucocorticoids are prescribed as anti-inflammatory drugs in the treatment of inflammatory disorders, asthma, allergic rhinitis, ulcerative colitis, ophthalmic, dermatological, neurological, autoimmune diseases, and hematological cancers [93]. Long-term use or high dosages of glucocorticoids for treating inflammation can cause muscle atrophy [94]. These drugs cause muscle atrophy by downregulating the rate of skeletal muscle protein synthesis and increasing protein breakdown [93,94]. In a relevant study, researchers examined the inhibition of cell growth and the induction of cell apoptosis by dexamethasone in C2C12 myoblasts to test the effects of quercetin [93]. To determine if quercetin is effective in protecting the C2C12 cells, scientists tested various concentrations of dexamethasone (0, 125, 250, 500, and 1000 μM) and quercetin (0, 25, 50, 75, and 100 μM); the dexamethasone was applied for 4 h and the quercetin for 24 h [93]. It was found that quercetin can be utilized to reduce dexamethasone-induced mitochondrial malfunction in C2C12 skeletal muscle cells by downregulating the Bax/Bcl-2 protein expression and reactive oxygen species (ROS) production, as well as restoring the ΔΨm imbalance [93]. It is evident that the results of this study demonstrate the ability of quercetin to reduce the effects of dexamethasone-induced skeletal muscle atrophy.

### 3.4. Hesperidin

Hesperidin is a naturally occurring flavanone glycoside present in citrus fruits and rosemary, with hesperetin as its aglycone form [33]. Hesperedin is a β-7-rutinoside of hesperetin and a disaccharide, rutinose [33]. To determine the effect of hesperidin, researchers evaluated 22–26-month-old mice with sarcopenia in comparison to 3–6-month-old mice. The mice were randomly assigned to five groups with 10 mice in each group: (1) young control mice, (2) young mice administered 10 mg/kg/day hesperidin, (3) old control mice, (4) old mice administered 5 mg/kg/day hesperidin (5) old mice administered 10 mg/kg/day hesperidin [95]. The study was conducted over 8 weeks, and it was determined that the older mice that were treated with hesperidin (5 mg/kg/day and 10 mg/kg/day) experienced increased muscle strength and a prevented decrease in grip strength [95]. Additionally, it was found that muscle size and mass increased in the quadriceps and gastrocnemius muscles in the hind limbs of the older mice [95]. The researchers also aimed to understand the mechanism involved in ameliorating sarcopenia. It was discovered that the administration of hesperidin proved effective in maintaining immune homeostasis by regulating the pro-inflammatory M1 macrophage and tissue repair-oriented M2 macrophage populations [95]. Hesperidin proved to be effective in treating sarcopenia, as this compound has the ability to maintain immune homeostasis through the regulation of macrophages and the inhibition of inflammaging.

### 3.5. Apigenin

Apigenin is a natural plant flavone that is abundant in Roman chamomile tea, parsley, celery, broccoli, bell peppers, and herbs [96]. Apigenin expresses anti-inflammatory and antioxidant properties [97]. In a relevant study, researchers hypothesized that apigenin supplementation relieves the effects of aging on skeletal muscle by enhancing its antioxidant properties and inhibiting hyperactive mitophagy and apoptosis [98]. To evaluate their hypothesis, the researchers compared 16-month-old mice (old group) and 6–9-month-old mice (young group). There was a total of sixty male mice: 48 old mice, and 12 young mice. The older mice were separated into four groups with 12 mice in each: Old control, standard chow diet and distilled water; Old + AP25, apigenin 25 mg/kg/day (low dose); Old + AP50, apigenin 50 mg/kg/day (middle dose); and Old + AP100, apigenin 100 mg/kg/day (high dose) [98]. The young mice (*n* = 12) represented a control group treated with a standard chow diet and distilled water [98]. The older mice showed a higher frailty index, reduced muscle cross-sectional area and weight, weaker grip strength, and a shortened running distance; however, apigenin demonstrated the ability to relieve muscle atrophy by inhibiting the loss of muscle mass and force [98]. Furthermore, it was shown that apigenin improves ATP content, enzymatic activities, and mitochondrial membrane potential, which aids in increasing mitochondrial function [98].

### 3.6. Catechins

Catechins are a type of flavonoid that can be found in unfermented green tea, black tea, coffee, berries, grapes, wine, and cocoa [37,38]. These types of flavonoids exhibit antioxidant, anti-inflammatory, antitumor, anti-microbial, anti-viral, anti-diabetic, and anti-obesity properties [38]. There is a group of catechins that are specifically found in tea which includes (−)-epigallocatechin-3-gallate (EGCG), (−)-epicatechin-3-gallate (ECG), (−)-epigallocatechin (EGC) and (−)-epicatechin (EC) [38]. A study conducted among older adults (65 years and older) with sarcopenia at the Harima Care Center in Hyogo, Japan tested a 24-week nutritional program that involved essential amino acid (EAA) and tea catechin (TCC) supplementation after resistance exercise (RE) to evaluate the condition of skeletal muscle mass [99]. The aforementioned group of catechins form the tea catechins utilized in the 24-week nutritional program. There was a total of 54 participants separated into three groups: RE (*n* = 18), RE with EAA supplementation (RE + EAA, *n* = 18), and RE with EAA and TCC supplementation (RE + EAA + TCC, *n* = 18). However, after separation into the experimental groups, eight participants withdrew, leaving RE (*n* = 15), RE + EAA (*n* = 15), and RE + EAA + TCC (*n* = 16). The exercise program occurred twice a week with an intake of 3000 mg of EAA and 540 mg of TCC via powder supplementation in mineral water [99]. The mean adherence rate for the 24-week nutritional program was 86.8% in the RE + EAA + TCC group, 86.4% in the RE + EAA group, and 85.4% in the RE group [99]. After the 24-week intervention, the results demonstrated that the RE + EAA + TCC group showed an increase in skeletal muscle mass (% Δ = 3.47), grip strength (% Δ = 7.18), knee extension strength (% Δ = 12.5), gait speed (% Δ = 2.56), and physical quality of life value (% Δ = 6.01) [99]. The skeletal muscle mass of the RE + EAA + TCC group was significantly higher than that of the RE group, but there was no significant difference in skeletal muscle mass between the RE + EAA group and the RE group [99]. This suggests that the combination of EAA and TCC after RE has a significant effect on skeletal muscle mass in older adults with sarcopenia.

As discussed above, catechins have proven to be effective in treating sarcopenia in the elderly population with regard to increasing skeletal muscle mass. In addition to tea catechins, high-flavonoid cocoa has also been tested as a supplement to aid in the treatment of sarcopenia. Cocoa is a natural food high in flavonoid concentration, with the most abundant flavonoid being epicatechin [37]. Due to the lack of knowledge regarding the effects of high-flavonoid dietary supplementation, most specifically epicatechin, on the elderly population, researchers in a recent study aimed to determine whether high-flavonoid cocoa improved markers for oxidative stress, inflammation, frailty, quality of life, and cardiometabolic health [37]. To examine the effects of the proposed hypothesis, the researchers conducted two studies with two population samples: the first study comprising middle-aged subjects (55–70 years old) and the second study comprising older subjects (65–90 years old) [37]. The first study was a 12-week double-blind, placebo-controlled trial where participants were assigned to consume the cocoa beverage in one of three experimental groups: (1) a cocoa-free, skim milk-based powder beverage (placebo); (2) an alkalinized natural cocoa powder without flavonoids (0 mg); or (3) a natural cocoa powder rich in flavonoids (179 mg) [37]. The second study tested the beverages with natural cocoa powder without flavonoids and natural cocoa powder rich in flavonoids in an older population for a shorter period (8 weeks). Coupled with the supplementation of the cocoa beverages, the participants were instructed to walk for 30 min/day. Results for the initial study showed improvements in oxidative stress, the skeletal muscle index, and quality of life [37]. Overall, in the initial study, the skeletal muscle index modestly increased, but the group treated with epicatechin experienced a significant increase by 0.8 ± 0.3 kg/m^2^ [37]. The results for the second study demonstrated significant improvements in metabolic, oxidative stress, and inflammatory endpoints along with improvements in physical performance, frailty indicators, and quality of life [37].

### 3.7. Curcumin

This is a compound derived from turmeric and has been shown to possess potent anti-inflammatory properties. Curcumin attenuates the expression of pro-inflammatory cytokines, such as tumor necrosis factor-alpha (TNF-α), interleukin-1 beta (IL-1ϐ), and interleukin-6 (IL-6) [39]. It achieves this by inhibiting the NF-kB pathway, the primary transcription factor responsible for the inflammatory cascades that catalyze the loss of muscle integrity [39]. The NF-kB pathway serves as a primary mediator of muscle loss by translocating into the cell nucleus and initiating the transcription of genes that encode muscle-atrophying proteins [39]. Once activated, this signaling cascade triggers a metabolic shift in which the rate of breakdown of muscle fibers exceeds the body’s ability to synthesize new tissue [100]. However, by preventing the nuclear translocation of NF-kB, curcumin reduces the inflammatory environment that drives the progression of accelerated muscle atrophy linked with aging [39].

## 4. Carotenoids with Therapeutic Potential in Sarcopenia

Carotenoids are lipid-soluble terpenoid pigments found in fruits, vegetables, algae, and other plant-derived foods that have attracted growing interest for their potential role in attenuating sarcopenia through modulation of oxidative stress, chronic inflammation, mitochondrial dysfunction, and impaired cellular stress responses [41]. Carotenoids primarily function as antioxidants and regulators redox-sensitive signaling pathways, particularly nuclear factor erythroid 2-related factor 2 (Nrf2) and nuclear factor kappa-light-chain-enhancer of activated B cells (NF-κB), which influence inflammatory cytokine production, mitochondrial integrity, and muscle protein turnover [40,101]. Beyond antioxidant activity, carotenoids may also influence autophagic regulation and mitochondrial quality-control pathways implicated in muscle aging, including AMPK- and Nrf2-associated signaling networks involved in cellular stress adaptation [102].

Structurally, carotenoids are divided into two major subclasses: carotenes, which consist solely of carbon and hydrogen atoms, and xanthophylls, which contain oxygenated functional groups that influence polarity, tissue distribution, and bioavailability [40]. Both subclasses exhibit antioxidant and anti-inflammatory activities, although their physiological effects may differ substantially depending on their molecular structure, absorption efficiency, and metabolic conversion. As discussed previously, carotenoid bioavailability is strongly influenced by the food matrix and lipid co-ingestion, factors that are particularly relevant in aging populations with impaired nutrient absorption [40].

At the observational evidence level, pro-inflammatory dietary patterns have been identified as quantifiable risk factors for sarcopenia. In a matched case–control study of 160 Iranian older adults, consisting of 80 sarcopenic individuals and 80 healthy controls, each unit increase in the Dietary Inflammatory Index was associated with a 27% increase in sarcopenia odds after multivariable adjustment, while each unit increase in the Dietary Inflammatory Score was associated with a 13% increase in sarcopenia odds. These findings provide an epidemiological rationale for examining carotenoids as anti-inflammatory dietary components capable of modulating the same inflammatory pathways implicated in skeletal muscle degradation, particularly NF-κB-mediated cytokine signaling and oxidative stress amplification [40,103].

### 4.1. Carotenes

Carotenes such as β-carotene, α-carotene, and lycopene are abundant in orange-pigmented and dark-green vegetables such as carrots, tomatoes, pumpkins, and sweet potatoes [40]. These compounds are primarily recognized for their ROS-scavenging capacity and ability to regulate inflammatory and redox-sensitive signaling pathways associated with skeletal muscle aging.

At the observational evidence level, α-carotene demonstrates the most specific association with muscle strength outcomes among individual carotenoids. In a cross-sectional analysis of 1172 older adults aged 50 to 85 years, serum concentrations of trans β-carotene, cis β-carotene, α-carotene, β-cryptoxanthin, lutein/zeaxanthin, trans-lycopene, vitamin E, and retinol were quantified by high-performance liquid chromatography, while muscle strength was evaluated using isokinetic knee extensor testing [42]. After adjustment for age, sex, protein intake, body mass index, C-reactive protein, physical activity, and additional confounders, serum α-carotene was the only antioxidant independently associated with greater muscle strength, whereas no significant associations were identified for the other carotenoids or vitamin E [42]. This specificity may indicate a distinct muscle-relevant biological activity associated with α-carotene rather than generalized effects that can be attributed to all carotenoids as a collective. Importantly, the cross-sectional design prevents causal inference, and serum antioxidant levels may also reflect broader dietary quality and health status.

Rather than focusing on single outcomes, studies evaluating carotene intake often highlight their contribution to overall antioxidant capacity and metabolic resilience in aging populations. In these analyses, higher carotene exposure tends to coincide with more favorable muscle-related profiles, suggesting that these compounds may help to maintain cellular environments that are less susceptible to oxidative damage and metabolic strain [104]. Although these findings are observational, they collectively point toward a potential role for carotenes in supporting muscle integrity by influencing physiological processes that decline with age.

Additional observational evidence is provided by the Framingham Offspring Study, which followed approximately 2400 adults with a mean age of 61 ± 9 years over approximately 12 years to evaluate dietary carotenoid intake in relation to longitudinal muscle outcomes [105]. Higher total carotenoid intake, including β-carotene and lycopene contributions, was associated with significantly attenuated declines in grip strength and gait speed, two clinically relevant diagnostic markers of sarcopenia. For every 10 mg/day increase in total carotenoid intake, annual grip strength decline was reduced by 0.0316–0.1223 kg/year, while gait speed decline was attenuated by 0.00008–0.0187 m/s/year in sex-combined analyses [105]. Although modest in magnitude compared with resistance-training interventions, these findings suggest that higher long-term carotenoid exposure may contribute to the preservation of physical function during aging. Mechanistically, these observations are consistent with the documented ability of β-carotene and lycopene to quench ROS, reduce lipid peroxidation, and suppress NF-κB-associated inflammatory signaling [40,101]. However, because carotenoid intake was analyzed collectively, the relative contribution of individual carotenes cannot be determined.

Despite promising epidemiologic associations, no completed randomized controlled trials using isolated α-carotene, β-carotene, or lycopene supplementation with sarcopenia-specific primary outcomes have been identified. Consequently, current evidence supporting carotenes remains observational and mechanistic rather than interventional.

### 4.2. Xanthophylls

Xanthophylls, including lutein and zeaxanthin, are oxygenated carotenoids primarily found in dark, leafy vegetables such as spinach and kale [40]. Compared with non-oxygenated carotenes, xanthophylls exhibit distinct tissue distribution and may accumulate preferentially within metabolically active tissues, where they contribute to antioxidant defense systems, stabilization of mitochondrial membranes, and the modulation of antioxidant enzyme activity [40,106].

At the observational evidence tier, lutein and zeaxanthin have been associated with the preservation of muscle-related functional outcomes. In the Framingham Offspring Study, higher lutein plus zeaxanthin intake was significantly associated with attenuated grip strength decline over approximately 12 years after adjustment for age, sex, physical activity, and total energy intake [β (SE) = 0.0316 (0.0146) kg/year per 10 mg/day intake] [105]. These outcomes are clinically relevant because declining grip strength represents a core diagnostic feature of sarcopenia. Nevertheless, the magnitude of association remained modest, with residual confounding related to overall dietary quality that cannot be excluded.

Further observational support was reported in a cross-sectional study of 2570 women aged 18 to 79 years, evaluating antioxidant vitamin and carotenoid intake in relation to sarcopenia-relevant phenotypes [104]. Total carotenoid intake, including xanthophyll fractions, was significantly associated with higher fat-free mass and improved lower extremity power. Differences between the highest and lowest intake quintiles ranged from approximately 1.0% to 7.5% depending on the measured endpoint [104]. Notably, associations were stronger in women younger than 65 years, suggesting that age-related reductions in carotenoid absorption, distribution, or tissue utilization may attenuate physiological responsiveness in older adults most vulnerable to sarcopenia. This finding highlights an important translational limitation because impaired bioavailability may reduce the effectiveness of dietary carotenoid interventions in advanced aging populations.

As with carotenes, evidence supporting xanthophylls remains largely observational. No completed randomized controlled trials using isolated lutein or zeaxanthin supplementation with muscle mass, strength, or physical performance as primary endpoints have been identified.

### 4.3. Carotenoid-Containing Extracts

At the preclinical mechanistic evidence tier, several carotenoid-containing extracts have demonstrated biological effects relevant to pathways implicated in sarcopenia pathophysiology, although none have been conclusively validated as anti-sarcopenic therapies. Importantly, these compounds have generally been evaluated in healthy animal models or non-sarcopenic systems rather than in established models of age-related muscle wasting. Consequently, their relevance to sarcopenia remains mechanistic and translational rather than clinically confirmed.

Astaxanthin is a xanthophyll carotenoid synthesized primarily by the microalga *Hermatococcus pluvialis* and subsequently accumulated in seafood such as salmon and crustaceans [40]. At the level of preclinical animal evidence, astaxanthin supplementation was evaluated in a 45-day controlled trial involving Wistar rats subjected to exhaustive swimming exercise [107]. Animals receiving 1 mg/kg/day astaxanthin demonstrated a 29% increase in time to exhaustion compared with controls (48.2 ± 14.1 min versus 37.4 ± 4.0 min). This improvement was accompanied by significant increases in skeletal muscle antioxidant defenses, including enhanced glutathione peroxidase activity, increased manganese superoxide dismutase activity, an 88% increase in glutathione content, and a 53% increase in protein thiol concentrations within soleus muscle tissue during exercise-induced stress. Additionally, ferric reducing antioxidant power increased by 62% following exercise in supplemented animals, whereas control animals demonstrated a 45% reduction [106]. These endpoints are mechanistically relevant to pathways implicated in mitochondrial dysfunction and oxidative injury associated with sarcopenic muscle degeneration. However, the study utilized healthy exercising rats rather than aged or sarcopenic models, limiting direct translational applicability.

Saffron (*Crocus sativus* L.) contains carotenoid-derived apocarotenoids including crocin and crocetin, which are biosynthetically derived from zeaxanthin [43]. At the mechanistic preclinical evidence tier, these compounds have demonstrated the modulation of multiple signaling pathways associated with oxidative stress and inflammation. Crocin and crocetin suppress NF-κB p50/p65 nuclear translocation, inhibit inducible nitric oxide synthase and cyclooxygenase activities, and activate the Ca^2+^/calmodulin-dependent kinase-PI3K/Akt-Nrf2 signaling pathways involved in antioxidant defense regulation [107]. These effects are accompanied by increased glutathione, glutathione peroxidase, catalase, and superoxide dismutase activity, as well as reductions in malondialdehyde concentrations, a validated marker of lipid peroxidation and oxidative damage [107]. Antioxidant responses appeared more pronounced at doses greater than or equal to 50 mg/day in available studies. Because elevated malondialdehyde and impaired antioxidant capacity are associated with age-related skeletal muscle dysfunction, these findings are mechanistically relevant to sarcopenia biology. However, no studies have evaluated saffron-derived compounds using sarcopenia-specific primary endpoints such as appendicular skeletal muscle mass, grip strength, or gait speed.

Tucumã (*Astrocaryum aculeatum*) is an Amazonian palm fruit containing carotenoids, flavonoids, triterpenes, and unsaturated fatty acids identified through phytochemical characterization [108]. Available evidence is currently limited to toxicological and antioxidant analyses conducted outside of skeletal muscle systems. Although antioxidant enzyme activity and oxidative stress modulation have been reported, no sarcopenia-specific mechanistic, animal, or clinical studies have evaluated tucumã in relation to muscle mass, muscle strength, or muscle decline [106]. Consequently, tucumã currently represents the weakest translational evidence among the carotenoid-containing extracts discussed.

At the human interventional evidence level, translational clinical research evaluating carotenoid-rich dietary patterns is beginning to emerge. The Villani randomized controlled trial protocol investigates whether increased consumption of carotenoid-rich fruits and vegetables combined with extra virgin olive oil can attenuate muscle loss during energy restriction in overweight and obese older adults aged 60 years and older [109]. This study was powered at 80% to detect significant changes in appendicular skeletal muscle mass, with a target enrollment of 73 participants assigned to either a high-carotenoid dietary intervention consisting of 375 g/day vegetables, 300 g/day fruit, and 40–60 mL/day extra-virgin olive oil or a lower-carotenoid control diet [109]. Appendicular skeletal muscle mass measured by dual-energy X-ray absorptiometry serves as the primary outcome, making this protocol one of the first clinical attempts to evaluate carotenoid-dense dietary patterns using a core sarcopenia diagnostic criterion. Outcome data have not yet been published, preventing the assessment of efficacy. Nevertheless, this trial represents an important translational step toward determining whether the observational associations between carotenoid intake and preserved muscle function translate into clinically measurable benefits under controlled conditions.

Collectively, current evidence suggests that carotenoids influence several biological pathways directly relevant to sarcopenia pathophysiology, although the strength of evidence varies substantially according to carotenoid subclass and study design. The strongest observational evidence pertains to higher total carotenoid intake and its association with an attenuated decline in grip strength, gait speed, fat-free mass, and lower extremity power [104,105]. Among individual carotenoids α-carotene demonstrates the most specific association with muscle strength outcomes in population-based analyses [42]. The strongest mechanistic preclinical evidence currently pertains to astaxanthin, which produced quanitifiable improvements in endurance performance and skeletal muscle antioxidant capacity in animal models [106], and to saffron-derived crocin and crocetin, which target NF-κB-, Nrf2-, and oxidative stress-associated pathways implicated in muscle degeneration [107]. In contrast, tucumã remains supported only by indirect antioxidant evidence without muscle-specific validation.

Despite these promising findings, no carotenoid or carotenoid-containing extract has yet demonstrated efficacy in a completed, adequately powered randomized controlled trial using muscle strength, muscle mass, or physical performance as sarcopenia endpoints. Consequently, current evidence remains insufficient to support formal clinical recommendations. Future research should prioritize standardized interventional designs, the evaluation of individual carotenoid subclasses rather than composite intake measures, the assessment of age-related bioavailability limitations, and the integration of carotenoid supplementation with resistance exercise and optimized protein intake within multimodal sarcopenia management strategies.

## 5. Organosulfur Compounds with Therapeutic Potential in Sarcopenia

Organosulfur compounds have attracted increasing scientific interest due to their potential role in mitigating sarcopenia and supporting skeletal muscle health [108,110]. These restorative processes are influenced by several cellular signaling systems. Oxidative stress responses are primarily regulated through the Nrf2-Keap1 axis, inflammatory signaling is moderated through NF-kB, and muscle protein degradation is primarily controlled by the ubiquitin-proteasome system, including FOXO-dependent activation of MuRF1 and atrogin-1 [100,110]. Through experimental systems, organosulfur compounds have demonstrated the ability to influence these pathways, primarily in preclinical models.

### 5.1. Sulforaphane

Sulforaphane is a bioactive isothiocyanate derived from cruciferous vegetables such as broccoli [110]. Sulforaphane is one of the most extensively studied dietary activators of the Nrf2-Keap1 antioxidant pathway. It modifies cysteine residues on Keap1, allowing for Nrf2 nuclear translocation and the upregulation of antioxidant enzymes such as superoxide dismutase, catalase, and glutathione-related proteins [108]. In skeletal muscle models, this activation is consistently associated with reduction in oxidative stress and improved redox buffering [111]. Beyond antioxidant signaling, sulforaphane has exhibited the ability to interact with inflammatory and catabolic pathways related to sarcopenia. In vitro and animal studies demonstrate the suppression of NF-kB activation, which plays a central role in cytokine-mediated muscle catabolism [100]. This is linked with downstream reductions in FOXO-mediated expression of muscle-specific E3 ubiquitin ligases, such as MuRF1 and atrogin-1, which control proteolysis through the UPS [94]. These findings remain primarily preclinical but demonstrate mechanistic consistency across multiple muscle-related models.

In SAMP8 mouse models of aging muscle, sulforaphane has been observed to preserve skeletal muscle architecture, reduce inflammatory infiltration, and maintain collagen and myofiber organization [111]. This suggests a functional preservation of muscle integrity under oxidative and inflammatory stress.

### 5.2. Allicin and S-Allyl Cysteine

Allicin and S-allyl cysteine (SAC) are organosulfur compounds derived from garlic (allium sativum), representing reactive and stable sulfur-containing forms. Allicin is formed through the enzymatic conversion of alliin upon tissue disruption and exhibits potent redox activity. In experimental models, allicin has been shown to reduce oxidative stress and attenuate muscle damage biomarkers such as creatine kinase following mechanical injury [45]. These effects have been primarily observed in acute animal and exercise-based human studies. SAC, a stable compound existing in aged garlic extract, demonstrates long-term antioxidant effects. It elevates glutathione levels, supports mitochondrial function, and provides stability to redox-sensitive organelles, specifically in cellular and animal models [112].

Together, these compounds are linked with reduced oxidative injury, improved recovery responses, and the mitigation of exercise-induced muscle stress. However, human data remain primarily limited to observational dietary associations rather than interventional studies.

### 5.3. Methylsulfonylmethane

Methylsulfonylmethane (MSM) is an organic sulfur compound involved in sulfur donation for amino acid synthesis and connective tissue maintenance. MSM contributes sulfur for the synthesis of cysteine and methionine, which supports the production of glutathione and the capacity of cellular antioxidants [113]. In experimental systems, MSM has demonstrated the ability to attenuate NF-kB-mediated inflammatory signaling, which leads to reduced pro-inflammatory cytokine expression and decreased catabolic stress in muscle tissue.

MSM has been observed in both animal and human studies, generally involving exercise and musculoskeletal stress. Human trials suggest reductions in inflammation and delayed muscle soreness, pointing toward functional recovery effects.

### 5.4. Ergothioneine

Ergothioneine is a sulfur-containing amino acid derived from dietary fungi and mushrooms [46]. Ergothioneine is transferred into cells through the OCTN1 transporter, allowing for selective accumulation in tissues with elevated oxidative demand, such as skeletal muscle mitochondria [114]. The chemical stability of ergothioneine in the thione form allows for sustained redox buffering devoid of rapid anti-oxidation. Mechanistically, ergothioneine defends against mitochondrial oxidative damage and instability of DNA, both of which are present in the age-related decline in skeletal muscle [114].

Preclinical studies have consistently demonstrated a reduction in oxidative injury and improvements in mitochondrial resilience. Human evidence consists primarily of dietary observational studies connecting mushroom consumption to favorable health outcomes.

Overall, organosulfur compounds demonstrate biologically plausible roles in modulating several pathways implicated in sarcopenia, specifically oxidative stress (Nrf2-Keap1), inflammatory signaling (NF-kB), and proteolytic regulation through the FOXO–UPS axis. Among these compounds, sulforaphane demonstrates the strongest and most consistent preclinical evidence for preserving muscle architecture and attenuating inflammatory degradation. Allicin, S-allyl cysteine, MSM, and ergothioneine similarly demonstrate antioxidant and anti-inflammatory properties related to muscle biology, although human interventional evidence remains limited.

## 6. Minor Phytochemical Bioactives with Therapeutic Potential in Sarcopenia

Emerging evidence suggests that several less-studied phytochemical bioactives may contribute to skeletal muscle preservation through antioxidant, anti-inflammatory, and mitochondrial regulatory mechanisms. Among these, trigonelline, a plant-derived alkaloid structurally related to nicotinic acid, has attracted attention because circulating levels decline in individuals with sarcopenia and correlate positively with muscle strength and mitochondrial oxidative phosphorylation [115]. Experimental studies demonstrate that trigonelline can enhance cellular NAD^+^ availability via the Preiss–Handler pathway, thereby [115] improving mitochondrial respiration, promoting biogenesis and attenuating age-related muscle wasting [115]. These findings support the broader concept that NAD^+^-boosting phytochemicals may help counteract age-associated skeletal muscle decline [116,117].

Additional alkaloids and terpenoids have also demonstrated anti-sarcopenic potential in precliniacal models. The β-carboline alkaloid norharmane, which is found in coffee, activates Nrf2-dependent antioxidant signaling through the p38 MAPK pathways, promoting mitochondrial biogenesis and reducing cellular senescence in skeletal muscle cells and aged mice [118]. Similarly, monoterpenes such as thymol, carvacrol, and camphene exhibit protective effects through the modulation of oxidative stress, autophagy, mitophagy, and muscle protein turnover [119,120,121]. These compounds help preserve mitochondrial quality control and reduce reactive oxygen species-mediated muscle atrophy [119,122]. Other plant-derived compounds further support the therapeutic potential of emerging phytochemicals in muscle health. Wheat seedling extracts improve muscle mass, strength, and protein content in aging models through activation of AMPK–SIRT3–PGC-1α signaling and the suppression of inflammatory cytokines such as TNF-α, IL-1, and IL-6 [90]. Likewise, lignans isolated from Schisandra species [48] have been shown to promote skeletal muscle cell proliferation and increase myosin heavy chain expression, suggesting potential regenerative benefits [123]. Collectively, these results highlight the growing therapeutic interest in emerging phytochemical compounds that target oxidative stress, inflammation, mitochondrial dysfunction, and impaired muscle regeneration in sarcopenia.

## 7. Translational Perspectives on Functional Recovery in Sarcopenia

Resistance exercise combined with optimized nutritional support remains the cornerstone of evidence-based sarcopenia management. Current clinical guidelines recommend resistance training (2–3 sessions/week) together with adequate protein intake (1.0–1.5 g/kg/day), leucine enrichment, and adequate vitamin D supplementation to improve strength, mobility, and physical performance [124,125,126]. These interventions consistently demonstrate clinically meaningful benefits in grip strength, gait speed, chair-stand performance, and frailty reduction, outcomes now considered more relevant than lean muscle mass alone for assessing sarcopenia progression and therapeutic response.

In contrast, pharmacologic therapies for sarcopenia remain limited. Although agents such as selective androgen receptor modulators and myostatin inhibitors (e.g., bimagrumab) have shown increases in lean body mass in phase II trials [127,128], improvements in muscle function and long-term safety and functional benefit have been inconsistent. Consequently, growing interest has focused on dietary phytochemicals—including polyphenols, flavonoids, terpenoids, and alkaloids—as adjunctive strategies capable of targeting multiple biological mechanisms underlying muscle aging [129,130].

Experimental and emerging clinical evidence indicates that phytochemicals such as resveratrol, quercetin, epigallocatechin gallate, and curcumin regulate pathways associated with muscle preservation (Table 2). Mechanistically, benefits are linked to the activation of PI3K/Akt and mTOR signaling pathways [75,131,132], the suppression of ubiquitin–proteasome-mediated proteolysis [133], the inhibition of NF-κB-driven inflammation [134,135,136], the enhancement of AMPK signaling and mitochondrial biogenesis (via PGC-1α) [137,138,139], and the preservation of satellite cell function (Pax7) [140,141,142]. Additional translational relevance includes the modulation of oxidative stress [138,143], gut microbiota composition [144,145], and epigenetic regulation (DNA methylation, histone modification, microRNA activity) [146]. However, despite a promising mechanistic rationale, current evidence for phytochemicals remains less robust than that for exercise and nutritional interventions due to variability in bioavailability, dosing, study duration, and endpoint selection. Importantly, phytochemicals may offer the greatest clinical utility when integrated with established interventions rather than used as stand-alone therapy. Emerging evidence suggests synergistic interactions between phytochemical supplementation, resistance exercise, and protein intake, particularly in older adults with metabolic dysfunction, frailty, or anabolic resistance. Future clinical trials should therefore prioritize multimodal intervention strategies and standardized functional endpoints, including muscle strength, mobility, balance, and quality of life, to determine the true translational value of phytochemical-based therapy in sarcopenia management.

## 8. Potential Drawbacks and Challenges

Despite promising mechanistic and emerging clinical evidence, several major challenges continue to limit the translational application of phytochemicals in sarcopenia management. One of the primary obstacles is poor bioavailability and unfavorable pharmacokinetic properties, as many compounds exhibit limited intestinal absorption, rapid metabolism, low tissue penetration, and fast systemic clearance, thereby reducing effective concentrations in skeletal muscle [39,153,154]. Additionally, formulation-related issues, including instability, low solubility, and variability in extraction and purification methods, further complicate clinical implementation and reproducibility across studies. Although novel delivery systems such as nano-formulations, liposomal encapsulation, and phytosome-based technologies may improve bioavailability, these approaches remain incompletely validated in older populations.

Another important source of variability is the gut microbiome, which plays a critical role in the metabolism, activation, and biotransformation of many dietary phytochemicals. Interindividual differences in microbiota composition may substantially influence therapeutic responsiveness, bioactive metabolite production, and clinical efficacy, particularly in aging populations with altered microbial diversity. In addition, older adults with sarcopenia frequently present with multimorbidity and polypharmacy, increasing the risk of drug–phytochemical interactions involving cytochrome P450 enzymes, transporter systems, anticoagulants, antidiabetic medications, and anti-inflammatory agents. These interactions may alter pharmacodynamics, safety profiles, and treatment outcomes.

Clinical translation is further constrained by the limited number of large-scale, long-term randomized controlled trials and by substantial heterogeneity in phytochemical dosing, intervention duration, extract standardization, and outcome definition. Importantly, improvements in lean muscle mass do not always translate into clinically meaningful gains in muscle strength, mobility, or physical performance. Regulatory challenges also remain significant as phytochemicals often occupy an uncertain position between dietary supplements and therapeutic agents, resulting in inconsistent quality control, labeling standards, and evidence requirements across regulatory agencies.

Finally, sarcopenia is a multifactorial disorder influenced by aging, inactivity, chronic disease, malnutrition, and metabolic dysfunction; therefore, phytochemicals alone are unlikely to provide sufficient therapeutic benefit without integration with established interventions such as resistance exercise, optimized protein intake, and broader lifestyle modifications. Future research should prioritize standardized formulations, precision nutrition approaches, biomarker-guided stratification, and rigorous multimodal clinical trials to better establish the efficacy, safety, and regulatory feasibility of phytochemical-based therapies for sarcopenia.

## 9. Future Perspectives and Conclusions

The experimental and clinical evidence reviewed here highlights the promise of phytochemicals as emerging therapeutic agents for sarcopenia. These plant-derived bioactive compounds target several biological processes implicated in muscle aging, including oxidative stress, chronic inflammation, and mitochondrial dysfunction. As detailed throughout this review, phytochemicals may help restore metabolic balance and support muscle maintenance during aging via the modulation of key signaling pathways such as PI3K/Akt-mTOR, AMPK–SIRT3–PGC-1α, NF-κB, and Nrf2 [75,90,131,132]. The pleiotropic nature of these compounds suggests that they may simultaneously target multiple interconnected drivers of sarcopenia, potentially offering advantages over single-target pharmacological interventions.

Despite these promising findings, several important translational challenges remain. A major near-term research priority is the establishment of standardized dosing strategies and treatment regimens, as substantial variability exists across studies with respect to compound formulation, dose, and duration. In parallel, future investigations should prioritize the validation of robust biomarkers for monitoring treatment response, including molecular, metabolic, inflammatory, and functional indicators of muscle health. Greater emphasis is also needed on pharmacokinetic and bioavailability studies, as many phytochemicals exhibit poor absorption, rapid metabolism, low tissue penetration, or variable biological activity depending on factors such as gut microbiota composition [48,49,54,58,155,156]. Advances in delivery technologies, including liposomal encapsulation and nanoparticle carrier technologies, may improve compound stability, bioavailability, and tissue-specific delivery [157].

Importantly, phytochemicals will likely be most effective as components of multimodal therapies, as opposed to stand-alone treatments. Future translational studies should therefore explore synergistic approaches that combine dietary bioactives with lifestyle-based interventions, such as resistance training and exercise, known to preserve muscle mass and function during aging. Additionally, there is a need for randomized controlled trials using standardized clinical endpoints to determine the efficacy, safety, and long-term therapeutic potential of these compounds in diverse aging populations.

In conclusion, phytochemicals represent diverse and biologically active compounds with considerable potential to modulate key molecular pathways underlying sarcopenia. While preclinical and early clinical findings are encouraging, further mechanistic research, pharmacokinetic characterization, biomarker validation, and rigorously designed clinical trials are necessary to identify optimal compounds, dosing strategies, and therapeutic contexts. Continued interdisciplinary research will be critical for translating phytochemical discoveries into meaningful therapies for preserving muscle health and functional independence in aging populations.

## Figures and Tables

**Table 1:** Classification of major phytochemicals ^1^.

Phytochemical	Subclass	Key Compounds in Sarcopenia Research	Natural Source
Polyphenols	Phenolic acids	Gallic Acid 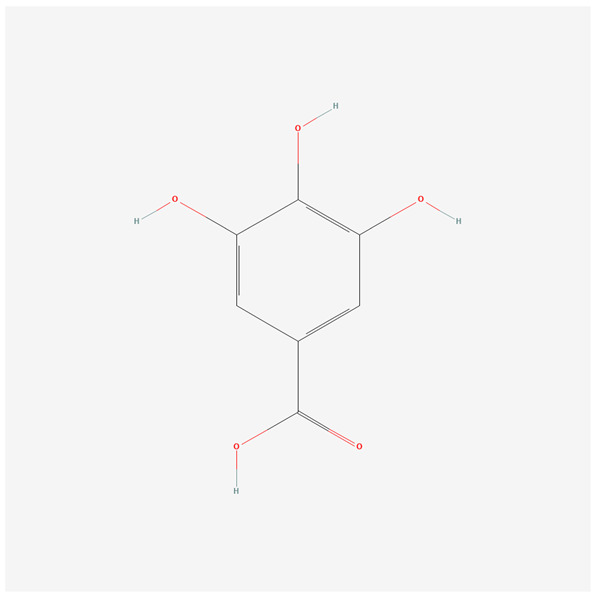 https://pubchem.ncbi.nlm.nih.gov/compound/Gallic-Acid	Tealeaves [25], apples, pomegranates, mangoes, pineapple, strawberries, raspberries, and citrus peels [26]
		Vannilic acid 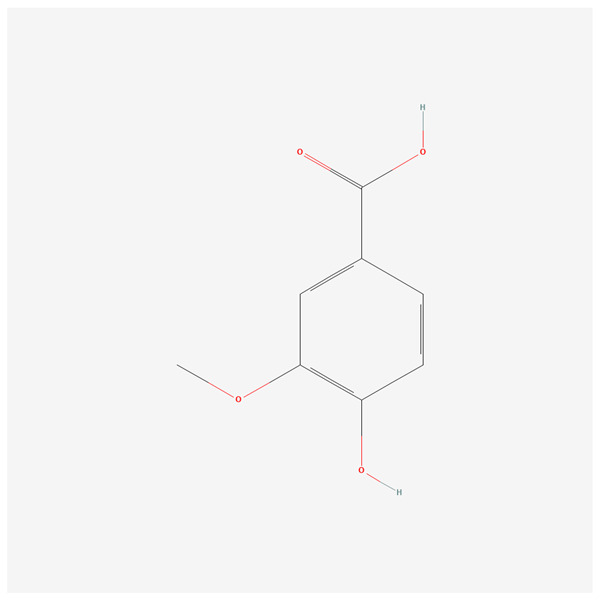 https://pubchem.ncbi.nlm.nih.gov/compound/Vanillic-Acid	Basil, oregano, rosemary, thyme, grains (rice and corn), date palm, olive trees, oranges, guavas, and cherries [27]
		Caffeic acid 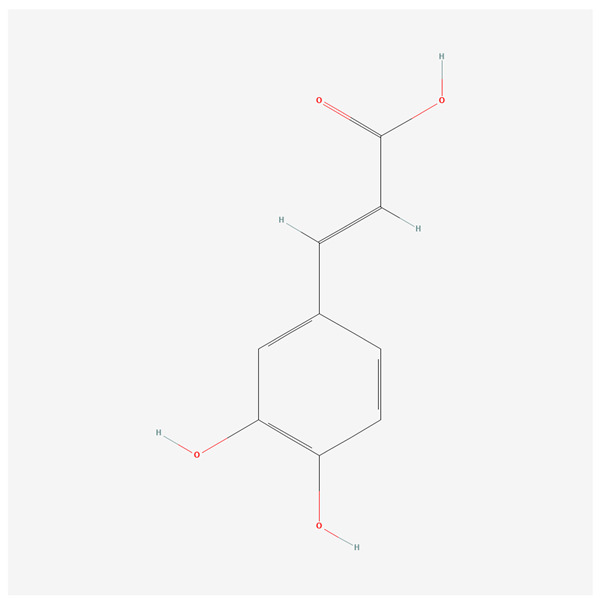 https://pubchem.ncbi.nlm.nih.gov/compound/Caffeic-Acid	Carrots, broccoli, and zucchini [28]
		Ferulic acid 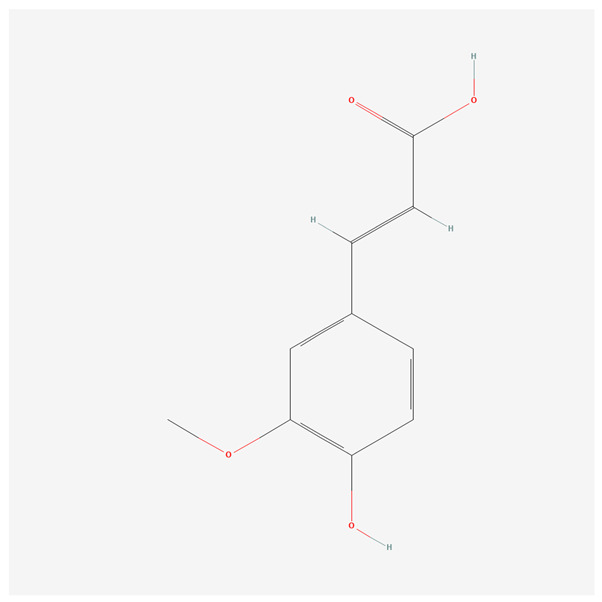 https://pubchem.ncbi.nlm.nih.gov/compound/Ferulic-Acid	Red beet, radish, pepper, turnips, and cucumber [28]
		Sinapic acid 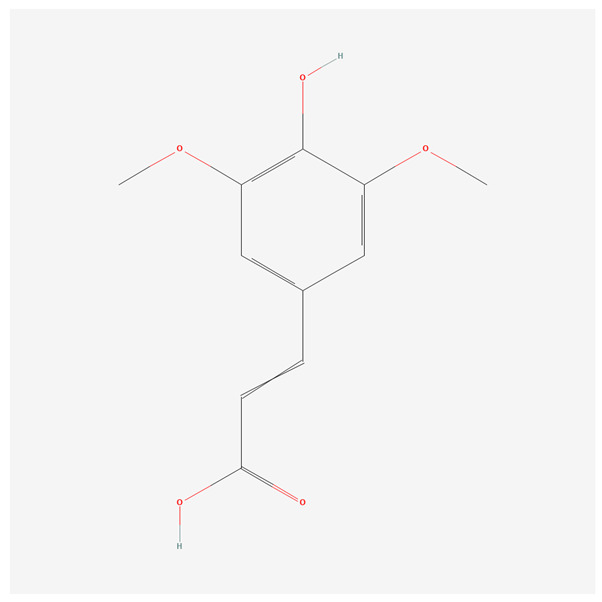 https://pubchem.ncbi.nlm.nih.gov/compound/Sinapic-acid	Broccoli, Chinese cabbage, cauliflower, turnips, white cabbage, and peas [28]
	Stilbenes	Resveratrol 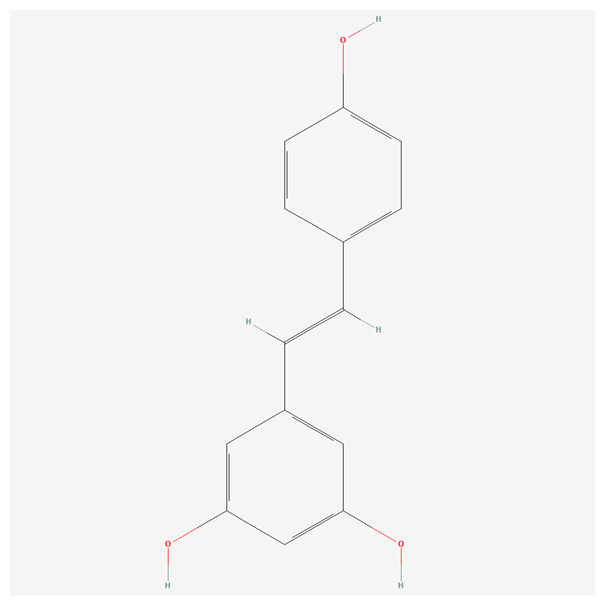 https://pubchem.ncbi.nlm.nih.gov/compound/Resveratrol	Grapes, lingonberries, blueberries, peanuts, and pistachios [29]
		Pterostilbene 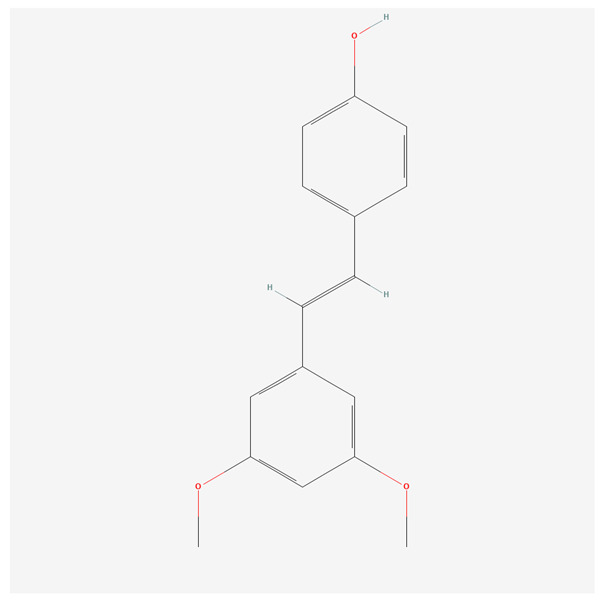 https://pubchem.ncbi.nlm.nih.gov/compound/Pterostilbene	Grapes and wine, [30]
	Flavonoids	Isoflavone 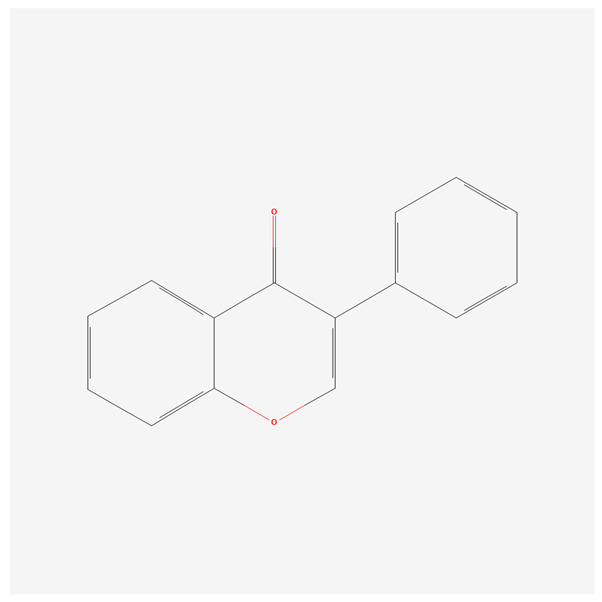 https://pubchem.ncbi.nlm.nih.gov/compound/Isoflavone	Soy [31]
		Sinensetin 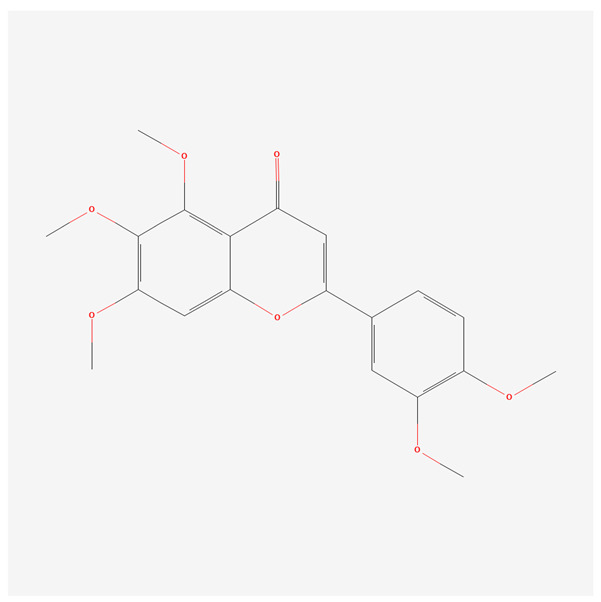 https://pubchem.ncbi.nlm.nih.gov/compound/Sinensetin	Citrus fruits [32]
		Quercetin 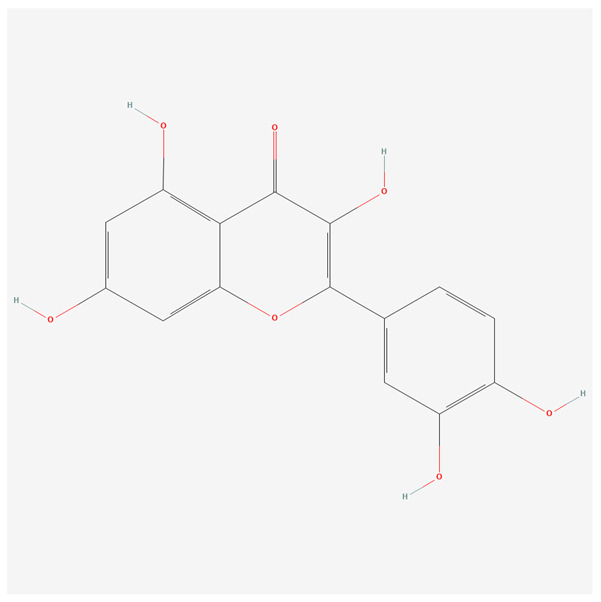 https://pubchem.ncbi.nlm.nih.gov/compound/Quercetin	Fruits, vegetables, herbs, and beverages
		Hesperidin 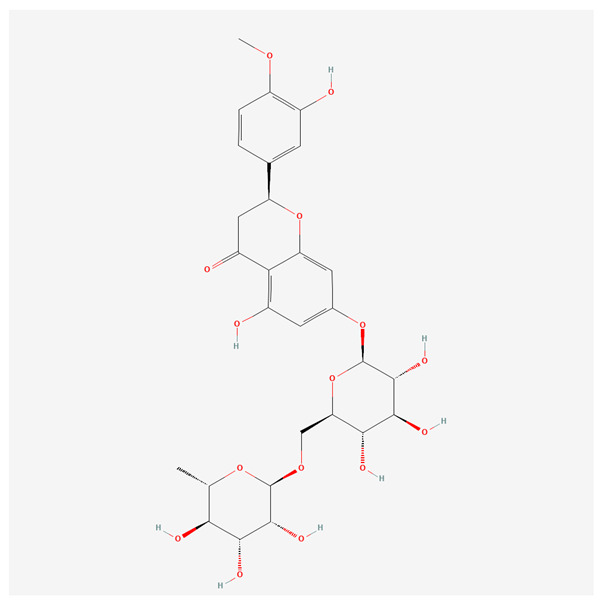 https://pubchem.ncbi.nlm.nih.gov/compound/Hesperidin	Citrus fruits [33] and rosemary [34]
		Apigenin 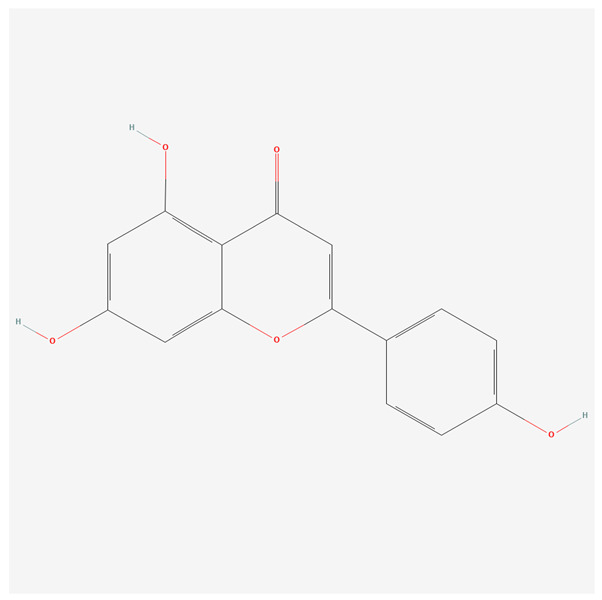 https://pubchem.ncbi.nlm.nih.gov/compound/Apigenin.	Roman chamomile tea [35], parsley, celery, broccoli, bell peppers and herbs [36]
		Epigallocatechin-3-gallate 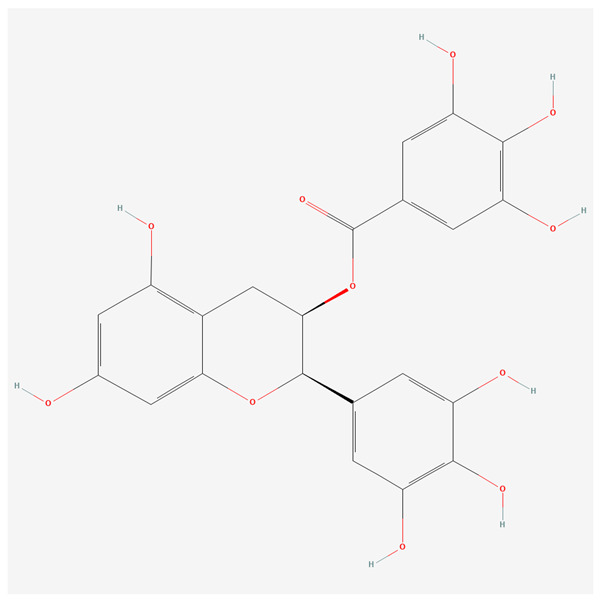 https://pubchem.ncbi.nlm.nih.gov/compound/Epigallocatechin-Gallate	Green tea, black tea, coffee, berries, grapes, wine, and cocoa [37,38]
		Epicatechin-3-gallate 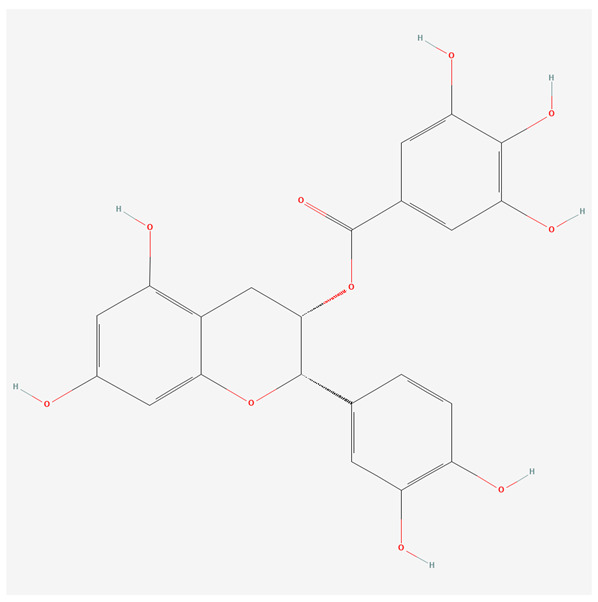 https://pubchem.ncbi.nlm.nih.gov/compound/Epicatechin-3-Gallate	
		Epicatechin 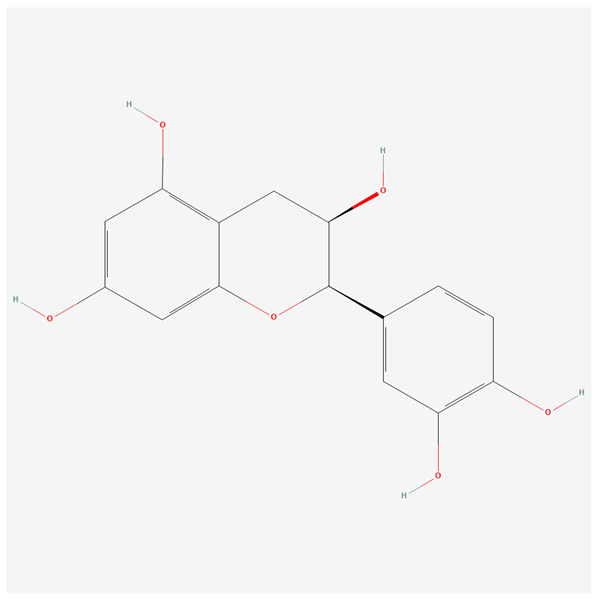 https://pubchem.ncbi.nlm.nih.gov/compound/Epicatechin	
	Curcuminoid	Curcumin 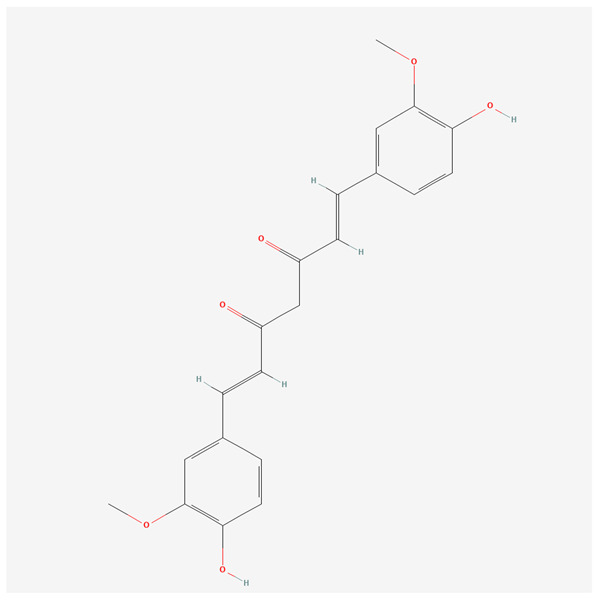 https://pubchem.ncbi.nlm.nih.gov/compound/Curcumin	Turmeric [39]
Terpenes	Carotenoids	α-Carotene 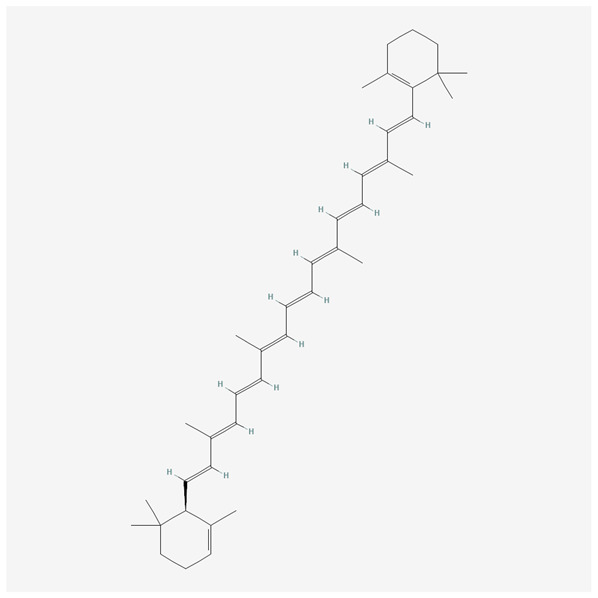 https://pubchem.ncbi.nlm.nih.gov/compound/Alpha-Carotene.	Carrots, pumpkin, sweet potatoes, winter squash, cantaloupe, mandarin oranges, apricots, green beans, broccoli, and peas
		β-Carotene 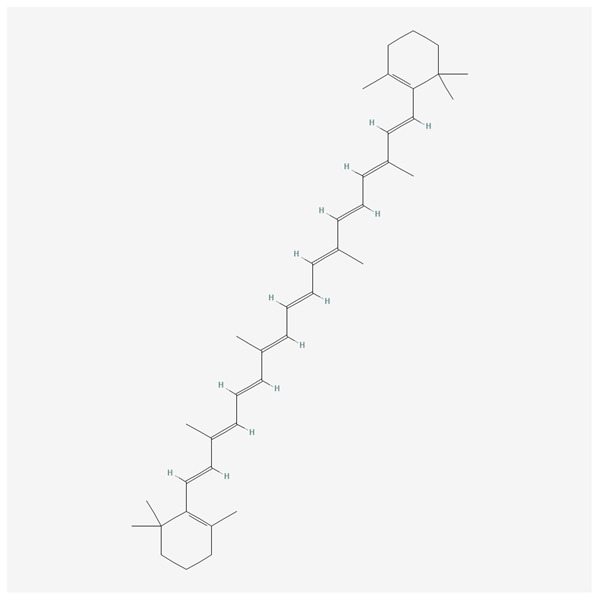 https://pubchem.ncbi.nlm.nih.gov/compound/Beta-Carotene	Carrots, sweet potatoes, pumpkin, butternut squash, dark leafy greens (kale, spinach), cantaloupe, red/yellow bell peppers, apricots, broccoli, and peas [40]
		Carotenoid 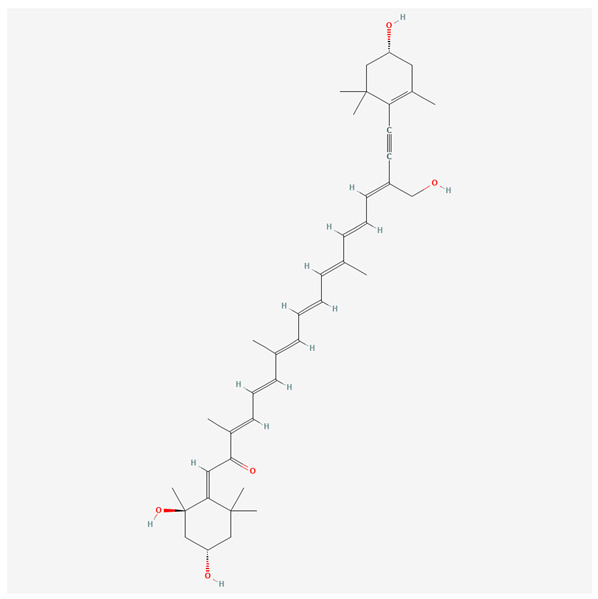 https://pubchem.ncbi.nlm.nih.gov/compound/Carotenoids	Orange, yellow, and red fruits and vegetables (carrots, tomatoes, and sweet potatoes, pumpkin, cantaloupe, bell peppers, mangoes), dark leafy greens (kale, spinach), and microalgae (*Dunaliella salina*, *Haematococcus pluvialis*) [41]
		Lycopene 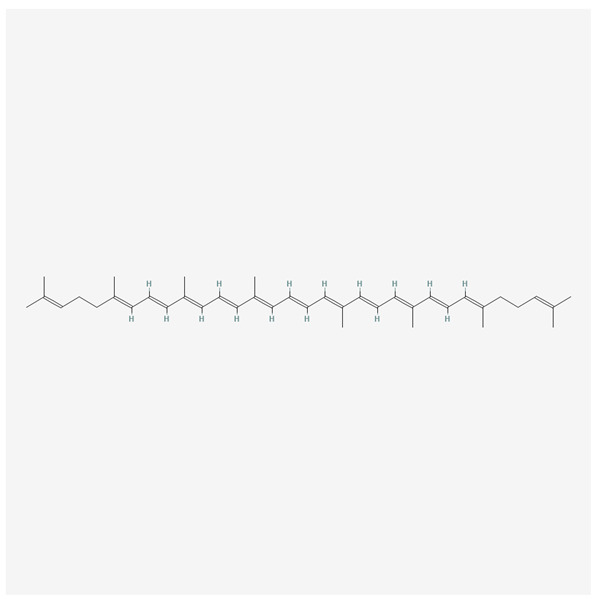 https://pubchem.ncbi.nlm.nih.gov/compound/Lycopene	Tomatoes (especially processed/cooked tomato products such as tomato paste, sauce, and juice), watermelon, pink grapefruit, guava, and apricots [40]
		Lutein 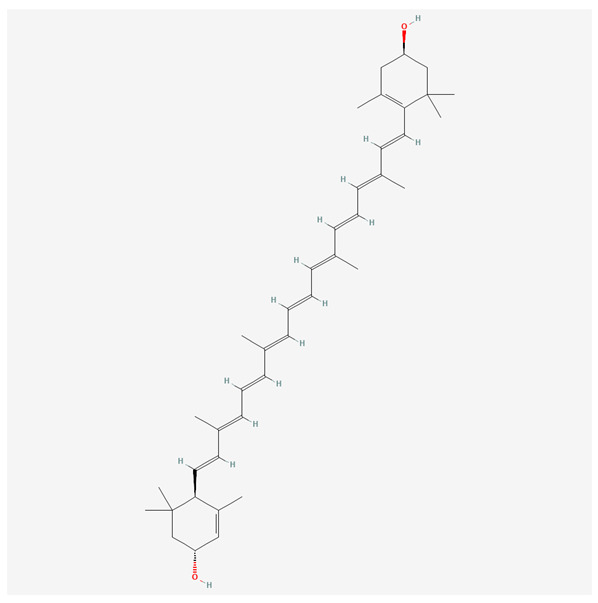 https://pubchem.ncbi.nlm.nih.gov/compound/Lutein-A	Dark leafy greens (kale, spinach, collards, turnip greens), broccoli, peas, summer squash, egg yolks, sweet yellow corn, avocados, and red peppers [42]
		Zeaxanthin 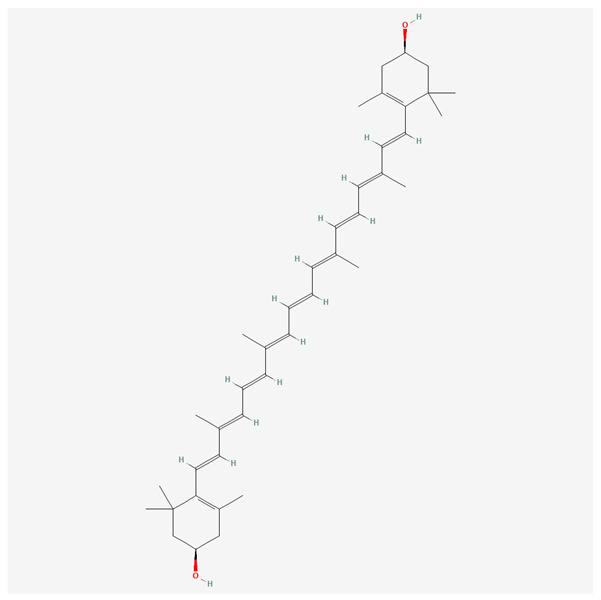 https://pubchem.ncbi.nlm.nih.gov/compound/Zeaxanthin	Dark leafy greens (kale, spinach, broccoli), corn, orange peppers, egg yolks, orange juice, honeydew melon, kiwi, and grapes [42]
	Tetraterpene	Astaxanthin 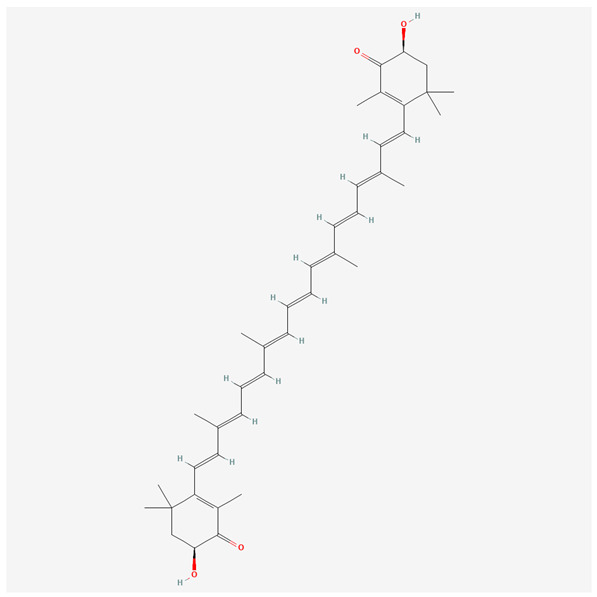 https://pubchem.ncbi.nlm.nih.gov/compound/Astaxanthin	*Haematococcus pluvialis* microalgae (primary natural source), wild salmon, rainbow trout, shrimp, lobster, crab, krill, and crayfish [40]
		Crocin 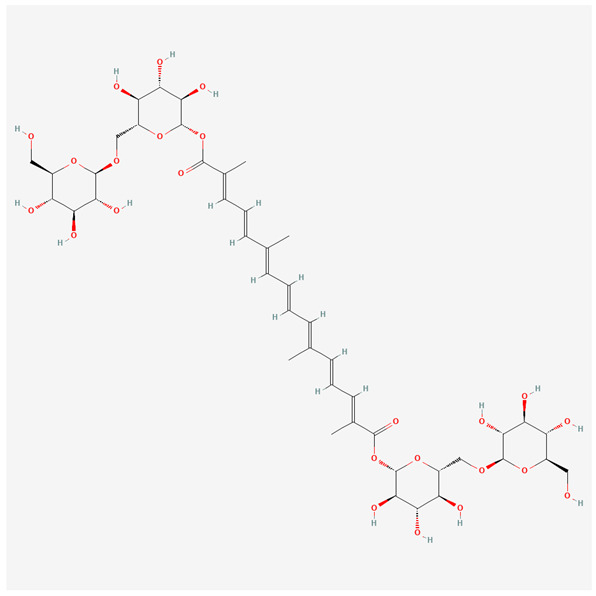 https://pubchem.ncbi.nlm.nih.gov/compound/Crocin	Saffron (*Crocus sativus*), gardenia fruit (*Gardenia jasminoides*), and *Perilla frutescens* [43]
		Crocetin 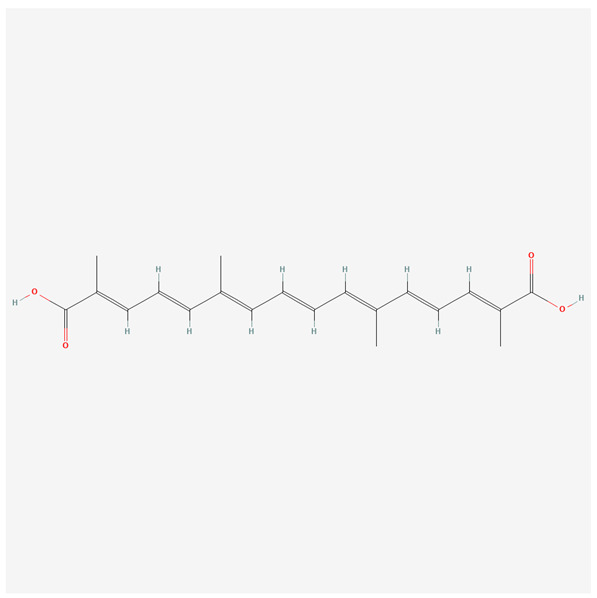 https://pubchem.ncbi.nlm.nih.gov/compound/Crocetin	
	Monoterpenes	Thymol 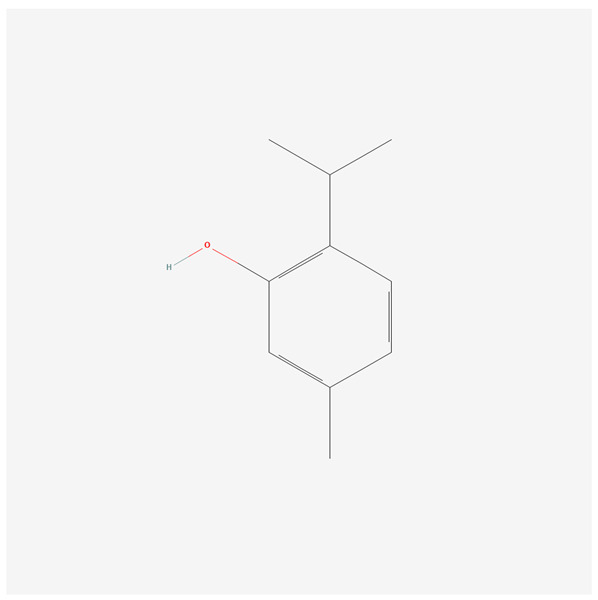 https://pubchem.ncbi.nlm.nih.gov/compound/Thymol	Thyme, oregano, and aromatic plant oils
		Carvacrol 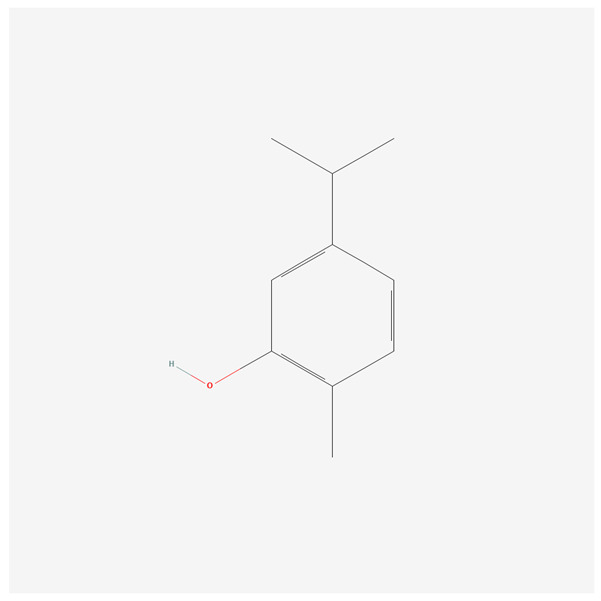 https://pubchem.ncbi.nlm.nih.gov/compound/Carvacrol	
		Camphene 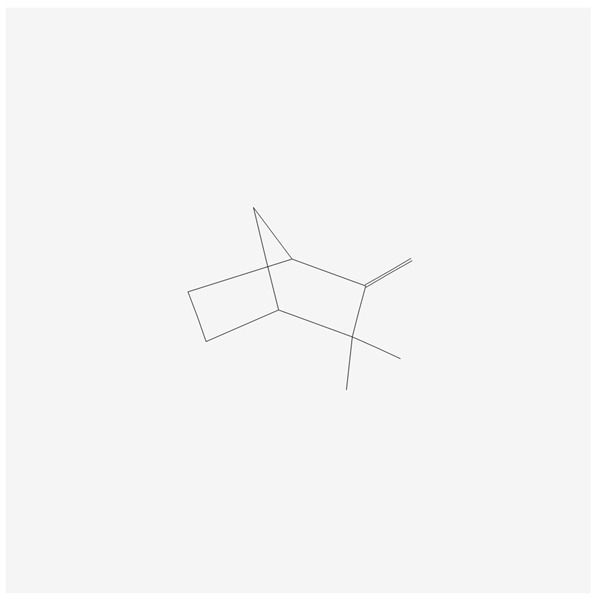 https://pubchem.ncbi.nlm.nih.gov/compound/Camphene	
Organosulfur Compounds	Isothiocyanate	Sulforaphane 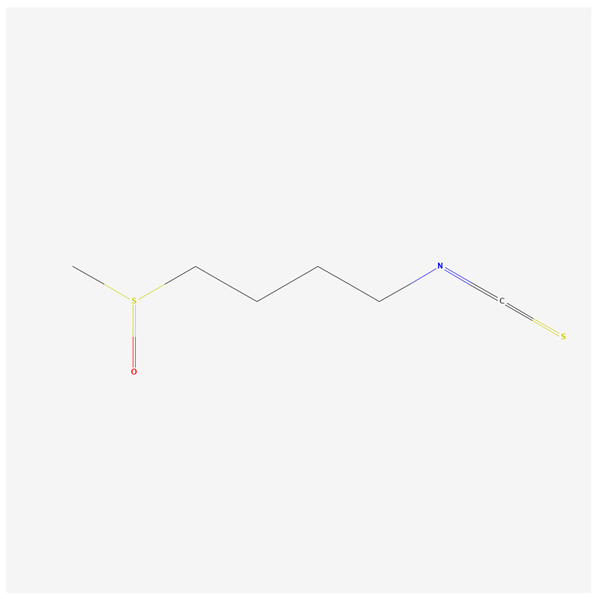 https://pubchem.ncbi.nlm.nih.gov/compound/Sulforaphane	Cruciferous vegetables, e.g., broccoli, cabbage, and kale [44]
		Allicin 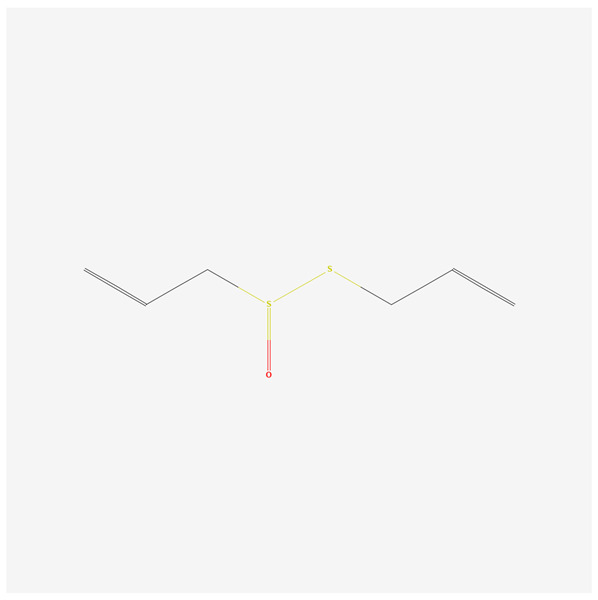 https://pubchem.ncbi.nlm.nih.gov/compound/Allicin	Raw bulb of garlic. Shallots, garlic chives, and wild leeks [45].
		S-Allyl Cysteine 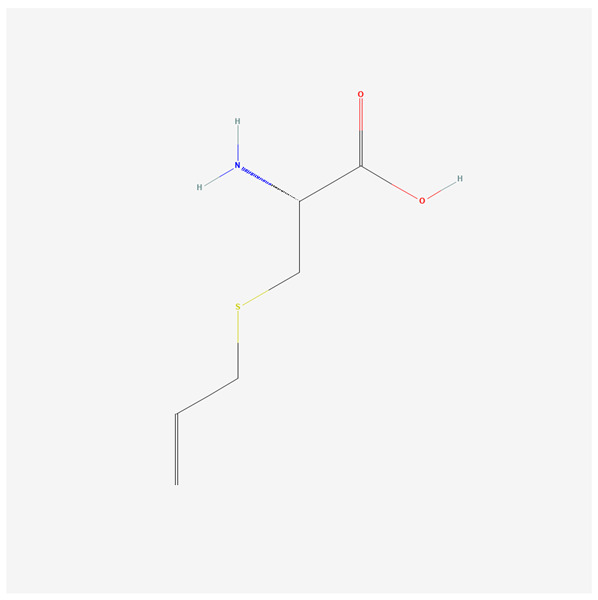 https://pubchem.ncbi.nlm.nih.gov/compound/S-Allylcysteine	Black (aged) garlic [45]
		Methylsulfonylmethane 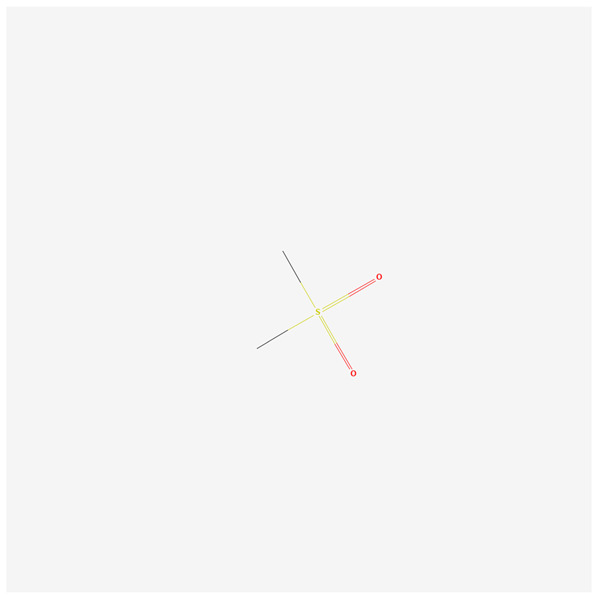 https://pubchem.ncbi.nlm.nih.gov/compound/Dimethyl-Sulfone	Green plants, algae, fruits and vegetables
		Ergothioneine 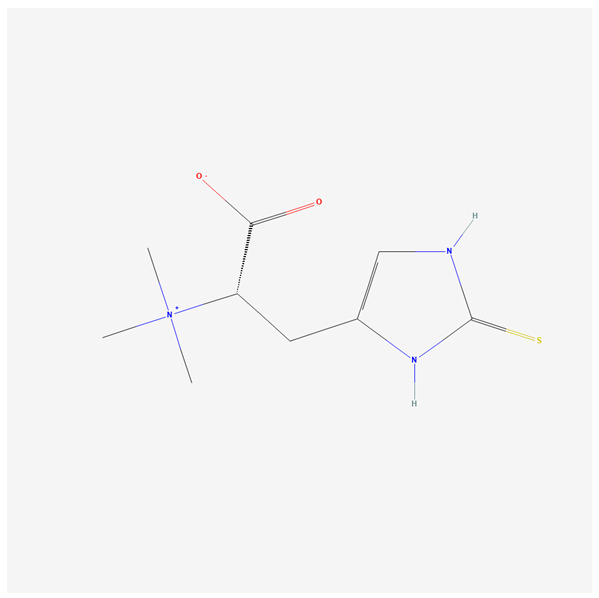 https://pubchem.ncbi.nlm.nih.gov/compound/Ergothioneine	Dietary mushrooms [46]
Alkaloids	Pyridine alkaloid	Trigonelline 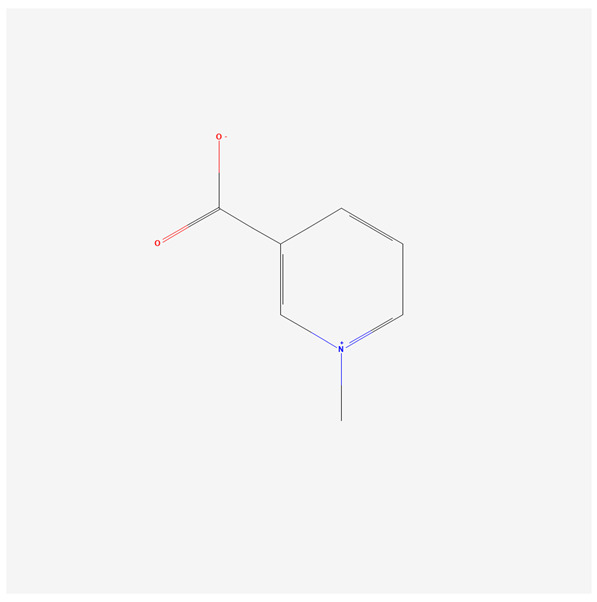 https://pubchem.ncbi.nlm.nih.gov/compound/Trigonelline	Fenugreek, coffee, etc. [47]
	Indole Alkaloid	Norharmane 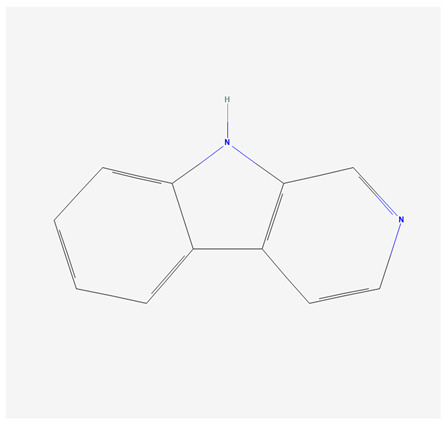 https://pubchem.ncbi.nlm.nih.gov/compound/Beta-Carboline	Coffee

^1^ All chemical structures are retrieved from the National Center for Biotechnology Information: https://www.ncbi.nlm.nih.gov/ PubChem Compound Summary from https://pubchem.ncbi.nlm.nih.gov/compound/ (accessed on 29 May 2026).

**Table 2:** Summary of randomized controlled trials on different sarcopenia interventions involving phytochemicals ^1^.

First Author, Year	Population	Intervention, Dose, and Sample Size	Phytochemical(s)	Endpoint	Outcomes
Aubertin-Leheudre et al., 2007 [147]	Post-menopausal obese-sarcopenic women (57–75 years old)	Isoflavone supplementation 70 mg/day (*n* = 12) vs. placebo (*n* = 6)	Isoflavones (Flavonoids)	24 weeks	Animal protein intake is associated with a better preservation of muscle mass index
Kim et al., 2016 [148]	Elderly women (over 70 years old) with sarcopenic obesity	Exercise only, exercise + EAA and TCC, EAA and TCC supplementation (*n* = 307)	Tea Catechin	12 weeks	Exercise plus EAA and TCC reduces body fat mass and improves muscle mass
Mungia et al., 2019 [37]	Male and female adults between 55 and 90 years old	Flavonoid rich mixture, alkalinized cocoa which eliminates flavonoid content, or placebo (*n* = 134)	Cocoa flavonoid-epicatechin	12 weeks	Regular flavonoids consumption positively affects blood oxidative stress and inflammation end points, and physical performance
Mafi et al., 2019 [149]	Older males (68.63 ± 2.86 years) with sarcopenia	Resistance training, epicatechin, and resistance training + epicatechin (*n* = 62)	Epicatechin	8 weeks	Resistance training combined with epicatechin improves muscle growth factors and prevents the progression of sarcopenia
Rondanelli et al., 2020 [150]	Older male and female adults with sarcopenia (≥65 years, 81 ± 6 years)	Supplementation with the experimental formula or Placebo (*n* = 140)	Protein-based nutritional formula enriched with leucine and vitamin D	4–8 weeks	Protein-based nutritional formula enriched with leucine and vitamin D improves physical performance and function, as well as muscle mass
Boutry-Regard et al., 2020 [151]	Older adults (60–90 years old) with mobility limitations	Electrical muscle stimulation 2×/week plus daily supplement: 20 g carbohydrate + placebo capsules (*n* = 12), 20 g whey protein isolate + placebo capsules (*n* = 15), or 20 g whey protein isolate + omega-3 fatty acids, rutin, and curcumin capsules (*n* = 10)	Rutin (polyphenol), curcumin (polyphenol), fish oil-derived omega-3 fatty acids (EPA, DHA)	12 weeks	Whey protein isolate plus bioactives capsules containing omega-3 fatty acids, rutin, and curcumin improves knee extension strength and gait speed
Tokuda et al., 2022 [99]	Older adults with sarcopenia (≥65 years)	Resistance exercise (RE) only, RE with essential amino acids (RE + EAA), and RE with EAA and tea catechins (RE + EAA + TCC) [*n* = 54]	Tea Catechin	24 weeks	Essential amino acids and tea catechin supplementation after resistance exercise improve skeletal muscle mass among older adults with sarcopenia.
Besora-Moreno et al., 2026 [152]	Men and women (69.6 ± 4.1 years) with probable sarcopenia	Refined olive oil (ROO; 30 mL/day; 90 mg caffeic acid) + maltodextrin placebo (7.5 g/day); EVOO (30 mL/day; 296–300 mg caffeic acid) + maltodextrin placebo (7.5 g/day); or (3) EVOO + prebiotic (EVOO + PREB; 30 mL/day + 7.5 g/day) [*n* = 38]	Phenolic compounds	12 weeks	Consuming phenolic-rich refined olive oil alone or combined with prebiotics improves muscle mass

^1^ All information in this table is based on the clinicaltrials.gov database.

## Data Availability

No new data were created or analyzed in this study. Data sharing is not applicable to this article.

## Abbreviations

The following abbreviations are used in this manuscript:

Akt	Protein Kinase B
AMPK	AMP-activated protein kinase
CHO	Preoperative Oral Carbohydrate
CRP	C-reactive protein
EAA	Essential amino acid
IGF-1	Insulin-like growth factor-1
FOXO	Forkhead box O
IL-6	Interlukin-6
IL-15	Interlukin-15
MAFbx	Muscle atrophy F-box
MSM	Methylsulfonylmethane
mTOR	Mechanistic target of rapamycin
MuRF	Muscle RING Finger 1
NF-κB	Nuclear Factor kappa B
Nrf2	Nuclear factor erythroid 2-related factor 2
PI3K	Phosphoinositide 3-kinase
PGC-1α	PPARγ Coactivator-1 alpha
PMFs	Polymethoxyflavones
ROS	Reactive oxidative species
SAC	S-allyl cysteine
SIRT3	Sirtuin 3
TCC	Tea catechin
TNFα	Tumor Necrosis Factor alpha
WPI	Whey Protein Isolate
